# Editorial: Ion Transport in Chloroplast and Mitochondria Physiology in Green Organisms

**DOI:** 10.3389/fpls.2016.02003

**Published:** 2017-01-05

**Authors:** Cornelia Spetea, Ildikò Szabò, Hans-Henning Kunz

**Affiliations:** ^1^Department of Biological and Environmental Sciences, University of GothenburgGothenburg, Sweden; ^2^Department of Biology, University of PadovaPadova, Italy; ^3^Institute of Neurosciences, CNRPadova, Italy; ^4^Plant Physiology, School of Biological Sciences, Washington State UniversityPullman, WA, USA

**Keywords:** ion transport, chloroplast, mitochondria, plant physiology, green organisms

This Research Topic represents a collection of articles either focusing on specific ion transport mechanisms or providing updated overviews of the research and transport mechanisms awaiting identification in chloroplasts and mitochondria. Some articles also cover detailed mechanisms of action and regulatory processes for already identified ion transport components. Other contributions unravel new possible players and interactions underlying the physiology of chloroplasts and mitochondria in plants, and green organisms in general.

Patch clamping of membranes is the most direct method used to characterize ion channel activities. Pottosin and Dobrovinskaya review the challenges in using this method for characterization of activities in chloroplast thylakoid membranes. Predominantly, these complications arise from the high protein/lipid ratio, the low phospholipid content, and presence of bulky ATP synthase subunits. All three aspects result in extremely fragile membrane patches. In addition, the authors discuss patch clamping of chloroplast envelope membranes together with the evidence for ion channel activities and identity of the genes involved.

Luesse et al. explore if loss of the mechanosensitive ion channels MSL2 and MSL3 in the chloroplast inner envelope interferes with common pathways for leaf development. MSL2 and MSL3 are required to maintain normal organelle size, shape, and ion homeostasis. Instead of constructing higher-order mutants, they apply RNA sequencing on *Arabidopsis msl2msl3* mutants and several other mutants similarly compromised in leaf morphology. Although the mechanism behind defective leaf morphology could not be solved, the authors generated a massive publicly available transcriptomics dataset, representing a valuable community tool for future studies on organelle dysfunction, ion homeostasis, and leaf variegation. The authors conclude that either RNA sequencing is not suitable to reveal the leaf developmental genetic network or that mechanisms determining leaf shape are even more complex than anticipated thus far.

The thylakoid-located putative Cl^−^ channel CLCe was hypothesized to play role in photosynthetic regulation based on initial analyses of *Arabidopsis* loss-of-function *clce* mutants (Marmagne et al., 2007). Herdean et al. reveal that the *clce* mutants are disturbed in chloroplast Cl^−^ homeostasis, leading to re-arrangements of the electron transport chain components in the thylakoid membrane. Moreover, while another thylakoid Cl^−^ channel was found important for fine-tuning photosynthesis in fluctuating light environments (Duan et al., 2016; Herdean et al., 2016), CLCe appears to function in the same process following dark adaptation. These findings speak for well-defined roles the two proteins may play in regulation of photosynthesis via Cl^−^ flux across the thylakoid membrane.

Höhner et al. review all currently known H^+^-dependent transport mechanisms in plastids. These carriers transport a variety of co-substrates from essential ions to other solutes, and impact several physiological processes, such as cation and pH homeostasis, osmoregulation, coupling of secondary active transport, and photosynthesis. Unfortunately, most activities are mediated by thus far elusive proteins, preventing exploitation of genetic tools to definitively prove their involvement in the above processes. More strikingly, also the existence of a potential H^+^ pump in the chloroplast envelope is still unclear, and thus should have high research priority in the future.

An extensive review on the role of ions in regulating light harvesting for photosynthesis is provided by Kǎna and Govindjee. The authors focus on the molecular interactions taking place at the negatively charged surface of the thylakoid membrane. This interaction results in so-called screening effects of the electric field on the membrane surface. Furthermore, they discuss the impact of ion-membrane interactions on thylakoid membrane stacking, state transition, and non-photochemical quenching. The review is dedicated to Jim Barber, who first recognized the importance of membrane surface charges for photosynthesis (Barber, 1980).

Transition metals such as iron, copper, and manganese are essential for chloroplast processes including photosynthesis. The review by López-Millán et al. deals with proteins involved in iron transport, storage, and assembly in the chloroplast as important players for homeostasis and photosynthetic performance. While the thylakoid iron transporter is still unknown, several systems function in acquisition of iron into the chloroplast across the inner envelope. In addition, the authors discuss the mechanisms for crosstalk between chloroplasts and mitochondria, both major control points of iron homeostasis.

The review by Aguirre and Pilon addresses the current knowledge about ATP-driven copper-transporters in chloroplasts, including envelope PAA1, and thylakoid PAA2, that work in concert to supply stroma and lumen with Cu ion. Insights into the regulatory mechanism of PAA2 are provided, such as the importance of miRNAs during low copper availability to prioritize delivery to plastocyanin. Since loss-of-function *paa1* and *paa2* mutants are suppressed by high copper levels in the growth media and the double *paa1paa2* is lethal, the authors suggest the possibility of mistargeting of PAA1 and PAA2 within the chloroplast, and the need for an alternative copper delivery route using low-affinity transport systems yet to be identified.

Three reviews on mitochondrial ion transport in plants are also part of this Research Topic. Carraretto et al. provide an update on the current knowledge about Ca^2+^ transport. Such knowledge is important because transient changes in Ca^2+^ concentration act as signals for transcriptional and metabolic responses, setting for an optimal performance of mitochondria. The molecular players involved in Ca^2+^ transport into mitochondria have been either identified or hypothesized.

Trono et al. review the knowledge about the mitochondrial ATP-dependent K^+^ channel discovered about 15 years ago in wheat (Pastore et al., 1999). This channel is compared to other known K^+^ channels, and the mechanism of regulating proton motive force and reactive oxygen species production in hyperosmotic stress is discussed.

Zancani et al. provide an updated overview of the permeability transition (PT) linked to programmed cell death in mitochondria, impacting plant development, and stress responses. Based on recent works suggesting that the mitochondrial ATP synthase functions as PT in mammals, the authors speculate that this could be the case in plants as well. Future studies are required to validate the role of mitochondrial ATP synthase as a PT channel in plant programmed cell death.

In summary, by analyzing loss-of-function and gain-of-function mutant phenotypes over the last years, exciting insights into the physiological significance of specific ions and the importance of their transport proteins in chloroplasts and mitochondria have been gained. However, more research is still required to identify the many elusive ion transport systems of these two fundamental organelles. Besides their initial discovery, studies of the transport mechanisms, structure/function, post-translational regulatory modifications, and connections to other transport proteins have great potential to improve our knowledge about ion transport in the physiology of chloroplasts and mitochondria, and may open new avenues for biotechnological applications, for instance to improve photosynthetic efficiency and stress tolerance.

## Author contributions

All listed authors made substantial contribution to the Research Topic. CS wrote the original version of the editorial. H-HK wrote and edited sections and contributed to the discussion. IS commented on the content. All authors read and edited the final version.

## Funding

Work in the authors laboratories was supported by the Swedish Research Council (grant no. 2016-03836, to CS), the Royal Society of Arts and Sciences in Gothenburg (CS), the Human Frontiers Science Program (grant no. 2015795S5W, to IS), PRIN of the Italian Ministry (IS), and by the National Science Foundation (NSF Career Award IOS–1553506, to H-HK).

### Conflict of interest statement

The authors declare that the research was conducted in the absence of any commercial or financial relationships that could be construed as a potential conflict of interest.
